# Synergistic Effect of 2-Acrylamido-2-methyl-1-propanesulfonic Acid on the Enhanced Conductivity for Fuel Cell at Low Temperature

**DOI:** 10.3390/membranes10120426

**Published:** 2020-12-15

**Authors:** Murli Manohar, Dukjoon Kim

**Affiliations:** School of Chemical Engineering, Sungkyunkwan University, Suwon, Kyunggi 16419, Korea

**Keywords:** 2-acrylamido-2-methyl-1-propanesulfonic, proton-exchange membrane, conductivity, cross-linking, temperature

## Abstract

This present work focused on the aromatic polymer (poly (1,4-phenylene ether-ether-sulfone); SPEES) interconnected/ cross-linked with the aliphatic monomer (2-acrylamido-2-methyl-1-propanesulfonic; AMPS) with the sulfonic group to enhance the conductivity and make it flexible with aliphatic chain of AMPS. Surprisingly, it produced higher conductivity than that of other reported work after the chemical stability was measured. It allows optimizing the synthesis of polymer electrolyte membranes with tailor-made combinations of conductivity and stability. Membrane structure is characterized by ^1^H NMR and FT-IR. Weight loss of the membrane in Fenton’s reagent is not too high during the oxidative stability test. The thermal stability of the membrane is characterized by TGA and its morphology by SEM and SAXS. The prepared membranes improved proton conductivity up to 0.125 Scm^−1^ which is much higher than that of Nafion N115 which is 0.059 Scm^−1^. Therefore, the SPEES-AM membranes are adequate for fuel cell at 50 °C with reduced relative humidity (RH).

## 1. Introduction

Recently, lots of polymer electrolyte membranes have been prepared from the sulfonation of aromatic polymers such as poly(arylene ether sulfone) [1,2,3,4] and modified poly(arylene ether sulfone) [5,6,7] for the application of electrodialysis and fuel cells as their rigid-rod backbone structures are basically quite stable in thermal and mechanical aspects. To optimize the membrane properties for fuel cell, huge efforts have been additionally given for their chemical and physical modifications such as grafting, [8] cross-linking, [9] degree of sulfonation, [10] surface modification [11,12,13,14,15,16,17], and composites [18,19,20,21,22].

Even though those sulfonated aromatic polymers have shown quite promising results [1,2,3,4,5,6,7] some critical shortcomings in membrane properties limited their wide commercialization. The proton conductivity of aromatic polymer membranes is generally lower than that of Nafion, a typical commercial polymer electrolyte membrane, because the aromatic polymer membranes usually form relatively smaller ion cluster dimensions for water flow in association with the lower acidic ion cluster environment. While the proton conductivity is possibly enhanced by an increment of the degree of sulfonation, it could sacrifice the mechanical and dimensional stability, as the membrane loses its toughness and accommodates too much water when the degree of sulfonating is beyond a certain level.

As one of the promising aromatic polymers, SPEES has been extensively studied for the application of the proton exchange membranes based on its outstanding thermal, mechanical, and chemical properties. However, as it also showed similar drawbacks, the achievement of higher proton conductivity crucially deteriorates other membrane properties. To short out these outcomes, we propose the AMPS introduced into SPEES in this study, because the highly concentrated sulfonic acid groups in the aliphatic AMPS are expected to enhance the conductivity and flexibility of the membrane [23,24,25].

As an aliphatic polar monomer, AMPS has been recently used for the preparation of polyelectrolyte for a few applications [22,23,24,25], because of its good thermal, chemical, and flexible properties. Polymerization of ionic liquid-AMPS (IL-AMPS) with a reactive surfactant produced polymers with high ionic conductivity suitable for the sensor, fuel cell, and battery applications [25,26,27,28,29,30,31,32,33,34]. The pristine PAMPS derivatives are, however, generally too weak in mechanical strength in water, illustrating hydrogel properties. The swelling ratio of poly(AMPS-*co*-MAA) hydrogels touches quite a high level with an increase of AMPS concentration because the sulphonic acid groups in AMPS are strongly hydrophilic [27,28,29].

In this term, we synthesized the hybrid aromatic-aliphatic polymer electrolyte using AMPS and SPEES. When such an AMPS was introduced to the slightly sulfonated SPEES, it will give an opportunity to optimize the synthesis of polymer electrolyte membranes with tailor made combinations of conductivity due to the distribution of AMPS in uniform way after the cross-linked with SPEES. In this study we synthesized the SPEES-AMs to investigate a variety of membranes properties including proton conductivity, thermal, chemical properties, water uptake, and swelling ratio. Fuel cell operation at low temperature will become a great deal in the PEMFCs with reducing the thermal energy for the application of low temperature fuel cell.

## 2. Materials and Methods

### 2.1. Materials

2-Acrylamido-2-methyl-1-propanesulfonic acid (AMPS), poly(1,4-phenylene ether-ether-sulfone) (PEES), TiCl_3_·AA and AlEt_2_Cl ([25] wt% in toluene) were purchased from Sigma-Aldrich (St. Louis, MO, USA). Methanol, acetone, *N,N*-dimethylacetamide (DMAc), *N*,*N*-dimethylformamide (DMF), tetrahydrofuran (THF), *N*-methylpyrrolidone (NMP), dimethyl sulfoxide (DMSO), sulfuric acid (H_2_SO_4_), were purchased from Daejung Reagents & Chemicals (Korea).

### 2.2. Synthesis of SPEES and Introducing of Thionyl Chloride into SPEES

**Scheme 1:**
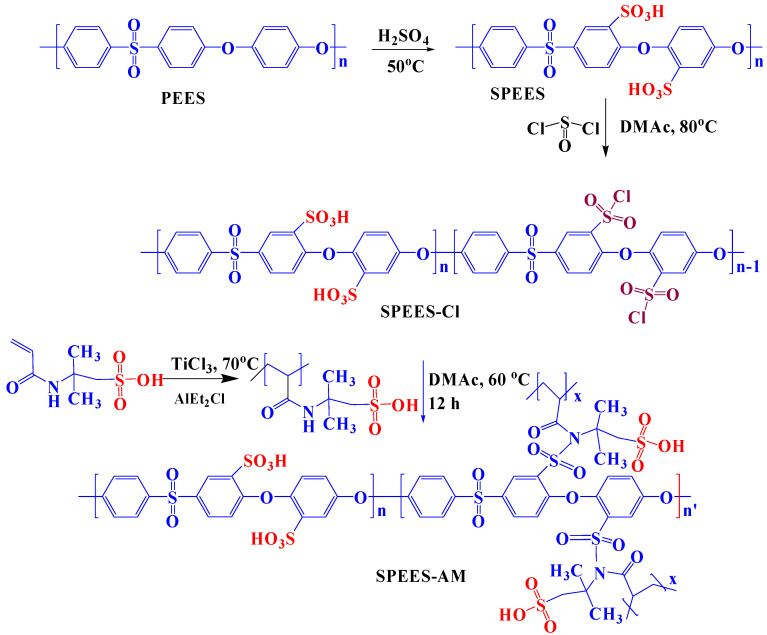
Synthesis scheme of 2-acrylamido-2-methyl-1-propanesulfonic (AMPS)-based SPEES-AM membrane.

The SPEES was synthesized [3,30], through the sulfonation of PEES at 50 °C for 8–9 h as shown in Scheme 1. The reaction product was precipitated in deionized (DI) water, and then washed several times with DI water to remove excess H_2_SO_4_. SPEES polymer was dried at room temperature in a vacuum oven for storage. The yield of the final SPEES product was 99%. To convert it to sulfonamide form [30,31], the required amount of SPEES was dissolved in DMAc and reacted with SOCl_2_ at 80 °C for 5–6 h to form the SPEES-Cl (Scheme 1). The product, SPEES-Cl, stems according to the above method and procedures.

### 2.3. Membrane Preparation

SPEES-Cl (1.0 g) was dissolved in DMAc (10.0 mL) under continuous magnetic stirring at 60 °C and AMPS was also dissolved in the same solvent. The AMPS solution was dropwise added to SPEES-Cl solution at different weight ratio of 1:0.1, 1:0.5, and 1: 1 AMPS pendant with benzene (SPEES-AM) under magnetic stirring for 12 h at 55–60 °C. The solution color was turned from colorless to light pale, and the color of pale increased with the AMPS volume.

Proton exchange membranes based on the aliphatic AMPS modified SPEES were synthesized using thermal copolymerization of AMPS and SPEES through sulfonamide formation, as shown in Scheme 1. After addition of TiCl_3_·AA and AlEt2Cl (25 wt% in toluene), the self-polymerization of AMPS [25,26,27,28,29] was conducted at 70 °C for 90 min, and then the reaction mass was quenched into methanol [32,33]. The product was filtrated and washed with methanol for several times and then dried at room temperature. The dry self-polymerized AMPS and SPEES were disloved in DMAc seperately. The AMPS solution was drop-wise added into SPEES solution and stirrered for 12 h at 60 °C. For membrane preparation, solution was casted onto the clean plane glass surface at 50 °C for 5–6 h.

### 2.4. Instrumental Characterization

^1^H NMR analysis of SPEES and SPEES-AM was performed using (NMR, Unity Inova Varian, Palo Alto, CA, USA) equipped with a 500 MHz high-resolution NMR console (S/N: S010002), a 51 mm Bore Oxford super conduction magnet (S/N: 70418) using dimethyl sulfoxide (DMSO-d6) as the solvent. Fourier transform infrared spectroscopy (FT-IR, Bruker, Germany) was used for the functional group investigation under the IR frequency range from 4000 to 600 cm^−1^. FE-SEM analysis has been tested for different membranes (surface, morphology) with JEOL, JSM 7000 F, Tokyo, Japan at 15.00 kV. Thermogravimetric analysis was performed with a thermogravimetric analyzer (TGA, TG7300, SEICO INST) for the thermal stability of the prepared membranes. The microstructure of the AMPS cross-linked membranes was analyzed by small-angle X-ray scattering spectroscopy (SAXS, Anton Paar^®^, Graz, Austria).

## 3. Physio-Chemical Characterization

### 3.1. Water Uptake and Swelling ratio and Ion-Exchange Capacity (IEC)

Membranes were dried in the oven at 120 °C until a constant weight marked as *Wdry*, and then immersed in H_2_O at room temperature for 24 h to reach the final dilution, the surface water was wiped with a filter paper to be immediately weighted, marked as *Wwet*. The water content (%) was calculated as the following Equation (1)

(1) $$\mathrm{WU}\;(\%)\;=\frac{Wwet-Wdry}{Wdry}\times 100$$

SIEC was calculated according to the previous report [3]. The swelling ratio (SR) was calculated with the *Ldry* and *Lwet* where *Ldry* is the weight and length of dry membrane and *Lwet* weight and length of wet membrane.

(2) $$\mathrm{SR}\;(\%)\;=\frac{Lwet-Ldry}{Ldry}\times 100$$

### 3.2. Oxidative, Chemical Stability, and Proton Conductivity

Oxidative stability was tested with Fenton’s reagent (3% H_2_O_2_ and 3 ppm FeSO_4_) for our prepared membranes at 80 °C in a closed vial for 72 h. Chemical stability was measured in the 3 M KOH and 3 M H_2_SO_4_ at 25 °C for one week. (Before all measurements, all samples dried under the vacuum oven at 80 °C for 3 days.)

The proton conductivity (σ) measured by the Equation (3):

(3) $$\sigma =\frac{L}{R\times W\;\times \;T}$$

where, *R* is the ohmic resistance, and *L* (= 0.425 cm) is the distance between the anode and cathode electrodes. *W* is the width, and *T* is the thickness of the membrane sample.

### 3.3. Membrane Electrode Assembly (MEA)

MEA was prepared for fuel cell performance. The catalyst ink was sonicated by a sonicator (Sonomasher, SL Science, Seoul, Korea) by adding 0.1 g of Pt/C (40%), 1 mL of DI water, 0.66 g of Nafion ionomer (5 wt% in IPA), and 8.042 g of IPA. The prepared solution was sprayed onto a gas diffusion layer (GDL) using a sprayer gun (Model GP-1, Tokyo, JAPAN 21701) in 5–10 min intervals. The MEA was prepared by pressing the catalyst-coated membrane using a heating press (Ocean Science, Seoul, Korea) at 100 °C and 5 MPa for 3 min. The active area of MEA was 6.25 cm^2^ and the Pt loading amount of both anode and cathode was 0.30 mg cm^−2^ each. The H_2_/O_2_ fuel cell was operated at 50 °C under 15% RH. The polarization curve for MEA fabricated with each membrane was obtained using a unit cell station (SPPSN-300) provided by CNL Energy (Seoul, Korea) at 200 cm^3^ min^−1^ H_2_ and O_2_ flow rate.

## 4. Results and Discussion

### 4.1. Strutucture Characterization

**Scheme 2:**
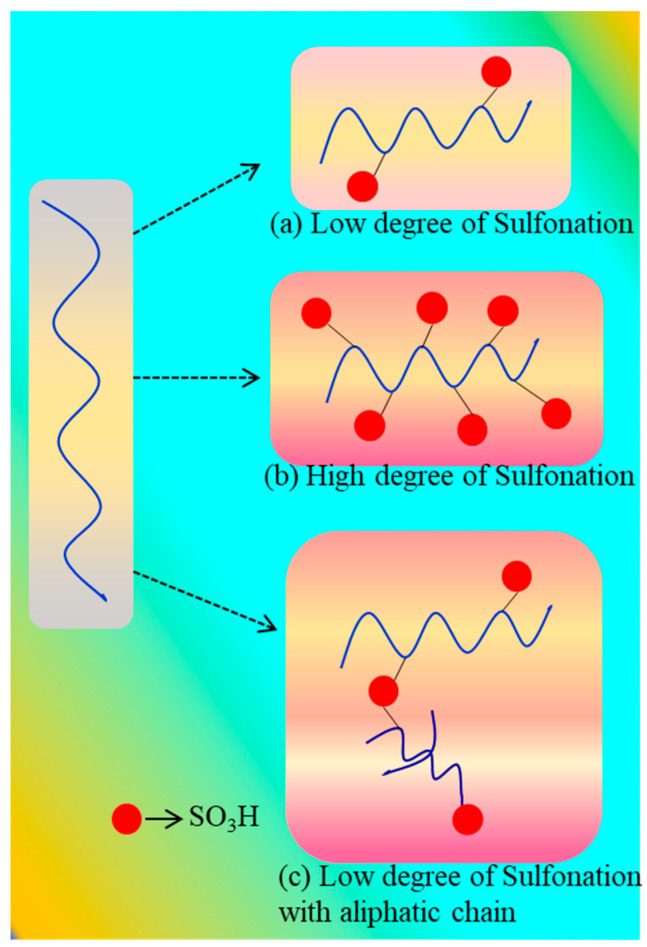
Schematic illustration of sulfonation of PEES (**a**) low degree of sulfonation (**b**) high degree of sulfonation and (**c**) low degre of sulfonation with apliphatic monomer.

Generally, the sulfonated aromatic-based polymer electrolyte membranes have a major disadvantage of the brittleness if the ionic site is directly attached to the polymer matrix (low sulfonation; Scheme 2a). These membranes exhibited low conductivity and poor fuel cell performance and if the degree of sulfonation increased, membrane matrix deteriorates (Scheme 2b). The detailed schematic illustration is presented in Scheme 2. Here in this present work, we applied the aliphatic monomer (AMPS) to reduce the problem of low sulfonation degrees (Scheme 2a), as presented in Scheme 1 and Scheme 2c.

**Figure 1:**
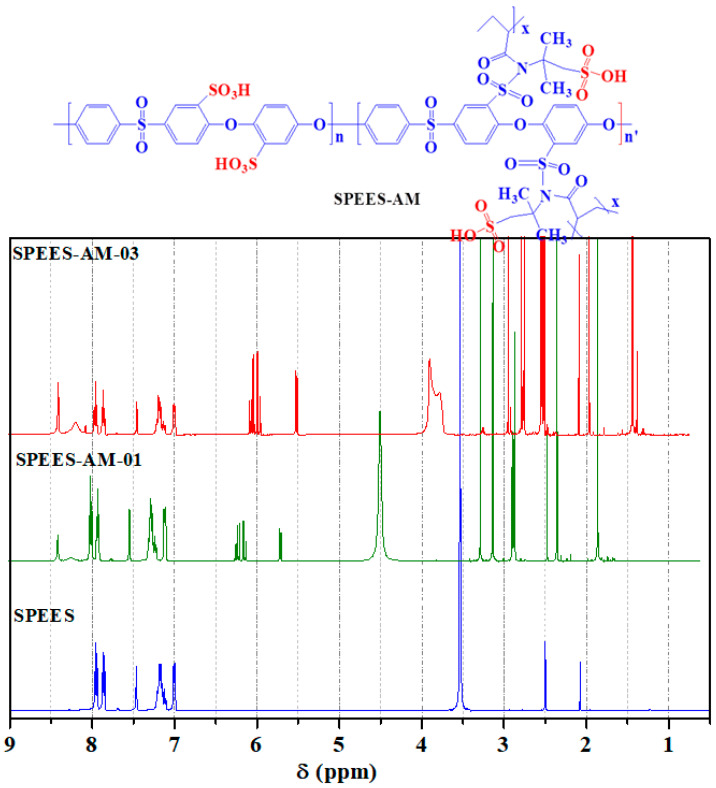
^1^ H NMR for SPEES, SPEES-AM-01, and SPEES-AM-03.

**Figure 2:**
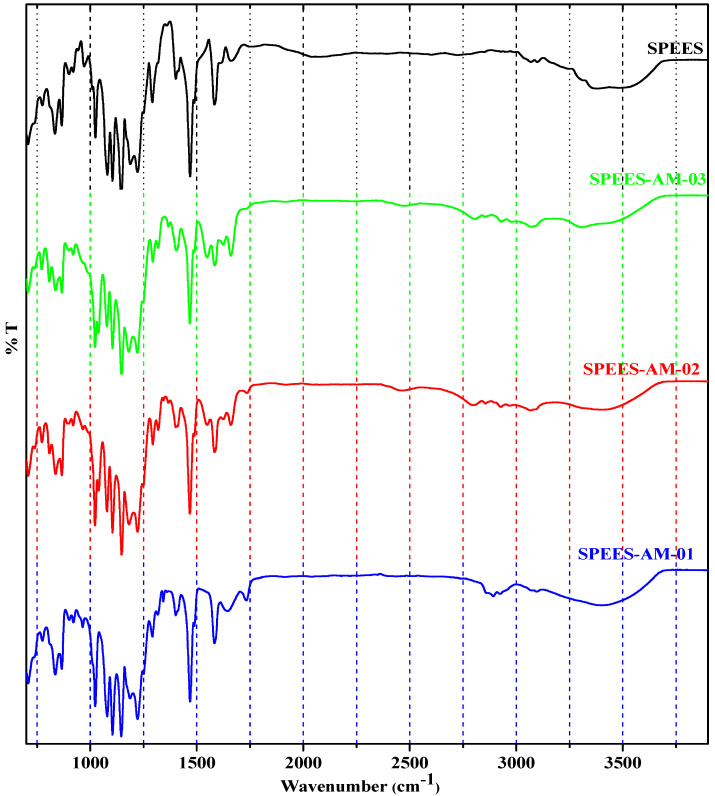
FT-IR for SPEES, SPEES-AM-01, SPEES-AM-02, and SPEES-AM-03.

^1^H NMR analysis confirmed the structure of SPEES and SPEES-AM. In Figure 1, the aromatic protons (Ar–H) are observed around = 7–8 ppm for SPEES, while the aliphatic protons are observed at δ= 1.5 ppm and 1.90 ppm, ((CH_3_)_2_ a) and another aliphatic –CH_2_-S at 2.80–2.90 ppm and CH=CH_2_ (multiplet, 5.50–6.20) for SPEES-AM. The peak observed at 4.5 ppm from N–H of AMPS (SPEES-AM-01) was shifted toward upfield (c, 3.7–4.00 PPM) (SPEES-AM-03 (FTIR spectra for SPEES and SPEES-AM are depicted in Figure 2. The aromatic C–C stretching vibration was observed at 1596–1471 cm^−1^ with medium to high intensity and the aromatic C–H, O=S=O (sulfone) group. The O–H group is observed at 3145–3025 cm^−1^, 1184–1139 cm^−1^, and 3250–3650 cm^−1^ (broad peak), respectively. The –SO_3_H group in SPEES is observed at 1247–1181 cm^-1^ in Figure 2. The cross-linking was confirmed from the presence of secondary sulfonamide group (1300–1350 cm^−1^). For SPEES-AM, C=C stretching vibration was observed at 1657 cm^−1^ and C=O was at 1727 cm^−1^. After AMPS grafting, the intensity of the O–H broad band decreased.

### 4.2. Morphological Structure

**Figure 3:**
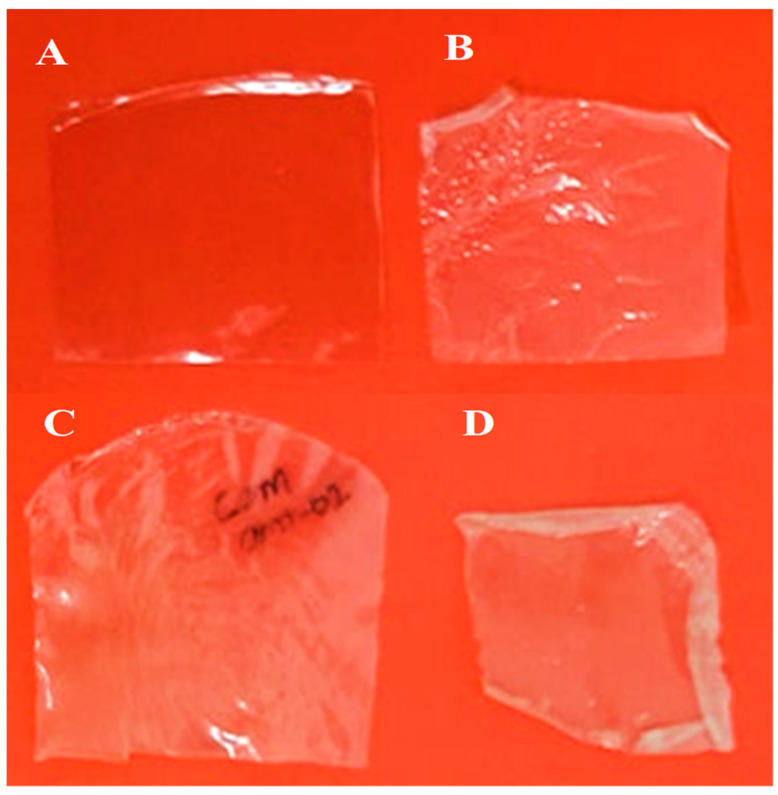
Optical images for (**A**) SPEES, (**B**) SPEES-AM-01, (**C**) SPEES-AM-02, and (**D**) SPEES-AM-03.

**Figure 4:**
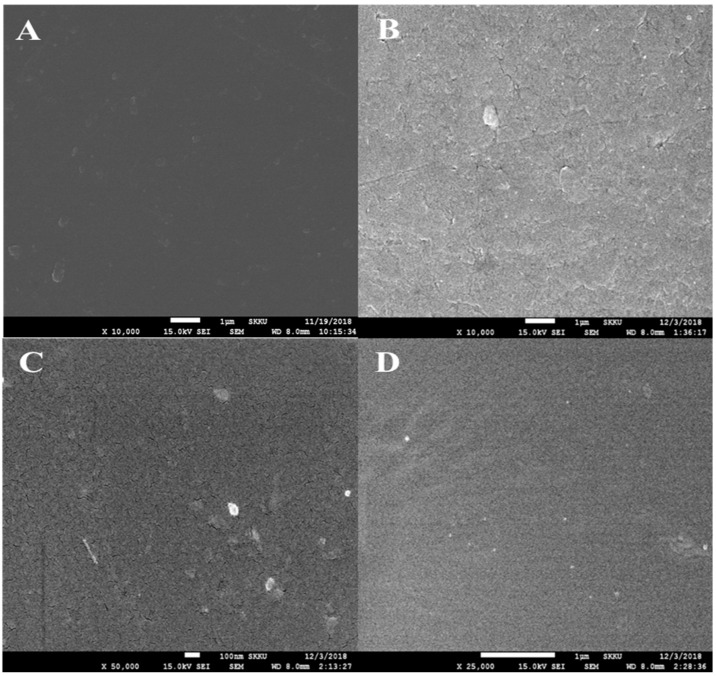
FE-SEM for (**A**) SPEES, (**B**) SPEES-AM-01, (**C**) SPEES-AM-02, (**D**) SPEES-AM-03.

While the SPEES membrane was transparent, SPEES-AM discolored the transparency into white haziness as shown in Figure 3A–D. All prepared membranes have been compatible with AMPS. In Figure 3D, SPEES-AM-03 membrane is still foldable after pressurizing without losing its primary stage. Figure 4 shows the SEM images of SPEES and SPEES-AMs.

**Figure 5:**
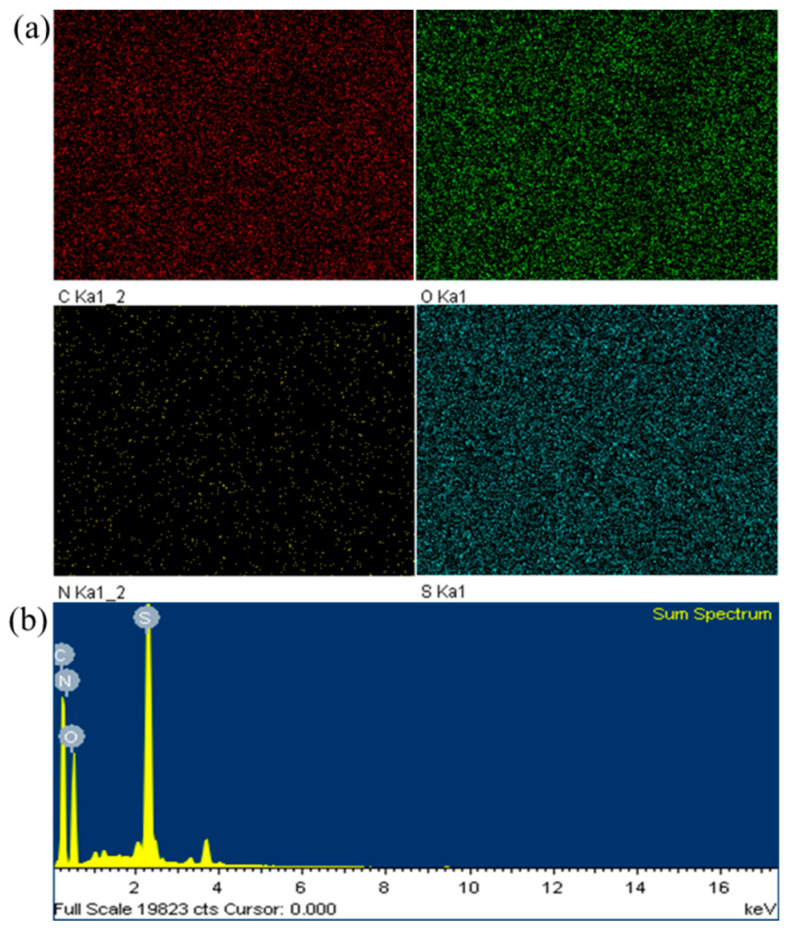
(**a**) Elemental mapping images for C, O, N, and S; and (**b**) EDS spectrum of SPEES-AM-02.

It can be seen that the SPEES membrane (Figure 4A) exhibits a smooth and tight surface while the SPEES-AM membrane (Figure 4A–D) exhibits an uneven and stiff surface, indicating that the phase-separated APMS domains are interconnected with SPEES phases. This difference is related to the different molar ratio of AMPS to SPEES in the membrane, and the obvious course and microporous morphology can be observed in the image of the membranes (Figure 4C,D). The images showed to be homogenous without any cracks and holes in the membrane phase. An AMP has higher hydrophilic nature because of higher ionic concentration than slightly sulfonated SPEES. The sulfonic groups can produce larger electrostatic repulsive forces than carboxylic groups [25,26,27], and thus the ion cluster size increased with increasing AMPS/SPEES molar ratio. Besides, the alkyl group of AMPS is hydrophobic, which can form hydrophobic micro-domains to decrease the hydrogen bonding interaction between hydrophilic polymeric chains and polymer backbone [29]. Element composition in the membrane matrix was analyzed by the EDS (Figure 5). EDS confirms the presence of C, N, O, and S via elemental mapping (Figure 5a) and also confirmed the homogeneous distribution of all presented elements, which suggest the successful synthesis of SPEES-AM and also presented the EDS spectra in Figure 5b.

**Figure 6:**
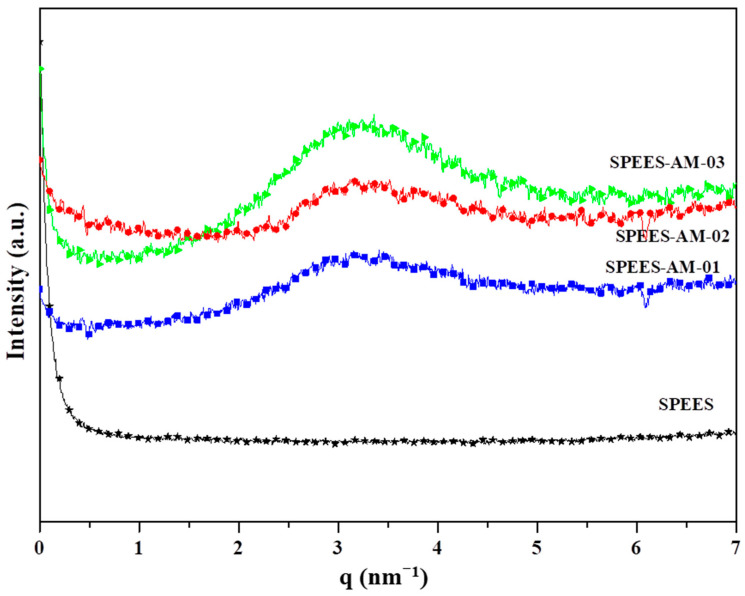
SAXS patterns of SPEES and SPEES- AM membranes.

SAXS measurements employed for the micro-phase separation of SPEES and SPEES-AMs. Since the location of the SAXS peak is related to the inter-cluster distance, the average dimension of the ionic clusters in the membranes, which constitute the ionic paths for proton migration in the PEMFCs, can be deduced from the SAXS data [7]. Figure 6 shows the SAXS patterns of the plain SPEES membrane and the SPEES-AM membranes. The average dimension of ionic clusters is calculated with the given Equation (4):

*d* *=* *2π*/*q* (4)

where, *q* is the scattering vector, which is equal to 44*π*/*λ*sinθ with 2θ being the scattering angle and *λ* the X-ray wavelength. As we can see that, in case of SPEES there is no micro-phase separation observed while in the incorporation of AMPS into SPEES increases the average dimension of the ionic clusters membranes showed distinct peaks at *q* = 3.2 nm^−1^, corresponding to a characteristic distance of *d* = 1.96 nm. All SPEES-AMs with more dense ionic groups tend to display a more profound scattering peak, which suggests larger spacing of ion-conducting channels. Generally, the polymeric electrolyte membranes form the ionic clusters from the counterbalance between the electrostatic energy released by ion-dipole interactions and the elastic free energy attributable to the deformation of backbone chain. This, combined with a higher concentration of –SO_3_H, provides membranes with high proton conductivity, makes us believe that the AMPS cross-linked SPEES membranes with reduced size of ionic clusters can exhibit fascinating properties for PEMFC applications at low temperature with reduced RH.

### 4.3. Thermal and Mechanical Properties

**Figure 7:**
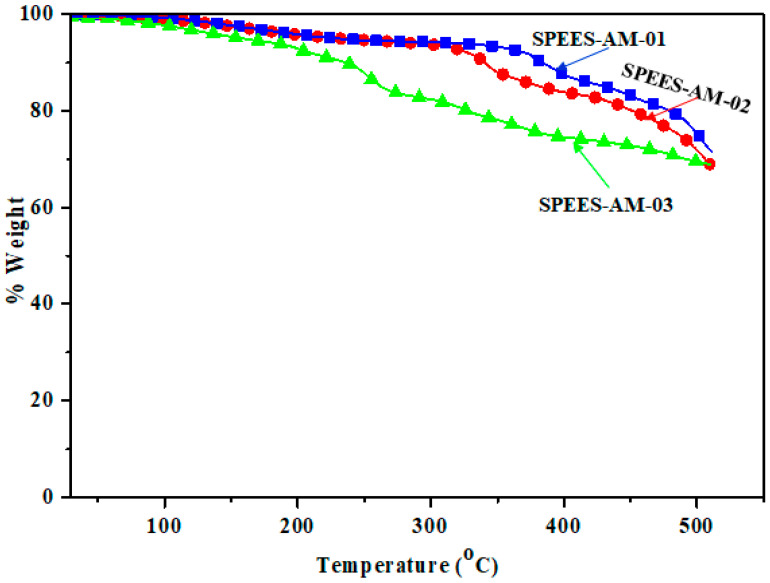
TGA for SPEES-AM membranes.

Thermal analysis of the prepared membranes showed three-step weight losses in the temperature range between 30 °C and 500 °C in Figure 7. All membranes were quite stable up to 100 °C. Beyond 100 °C, the first weight loss was observed by the evaporation of bound water in the membrane via ionic charge of –SO_3_H. SPEES-AM-03 showed the maximum weight loss in this region because of the presence of more bound water associated with more functional groups in it. The second weight loss was observed around 200–350 °C because of the decomposition of the sulfonic acid groups in SPEES-AM membranes. Weight loss up to 350 °C for the SPEES-AM-03 membrane was appreciably higher than those for the SPEES-AM-01 and SPEES-AM-02 membrane because more sulfonic acid groups were present inside it. The third weight loss step was attributed to the decomposition of the polymer backbone around 350 °C in SPEES-AM membranes.

### 4.4. Water Uptake and Swelling Ratio and IEC

The water uptake and swelling behavior of the membranes are key factors that significantly affect the proton conductivity, and mechanical properties of membranes. It can be clearly seen that the water uptake increased with the increment of AMPS content because of more sulfonic groups incorporated within the SPEES-AM membranes [27,28,29]. The effect of AMPS content on water uptake, the swelling degree, and IEC was studied and summarized in Table 1 and the solubility test is also presented in Table 2.

As the concentration of AMPS increases the swelling ratio increases because of the higher concentration of -SO_3_H groups which are surrounded by a large number of water molecules thereby increasing the swelling capacity. It can be seen that SPEES-AM-01 exhibited minimum water uptake and swelling at low temperature of 30 °C in comparison with SPEES-AM-02 and 03. IEC value also increased with increasing amount of functional group from 1.75 to 2.17 (meq·g^−1^).

### 4.5. Oxidative and Chemical Stability

**Figure 8:**
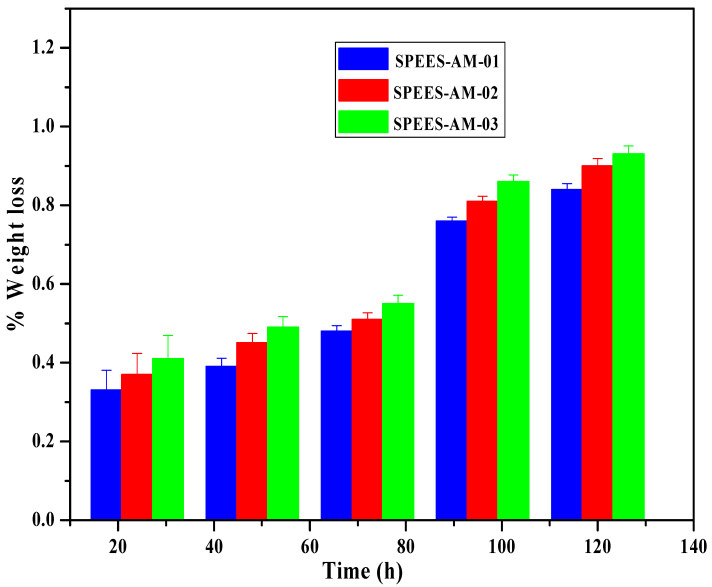
Oxidative stability test for SPEES-AM-based membranes at 60 °C.

The oxidative stability of SPEES-AM membranes has been measured as an important property of esteemed membranes related to durability in Fenton’s reagent (3% H_2_O_2_ and 3 ppm FeSO_4_). Figure 8 defines the radical degradation of AMPS-based membranes measured in Fenton’s reagent at 60 °C. The degradation during the oxidative testing of the membranes is estimated by the weight loss, conductivity loss, and mechanical loss. As shown in Figure 8, the membranes show good oxidative durability in Fenton’s solution. The SPEES-AM-01 membrane was observed to lose approximately ~1.5% weight after being immersed in a 3% H_2_O_2_ solution for 120 h. An initial sharp decrease in weight percentage of SPEES-AM membrane between 0.42 and 0.53% was observed after 72 h of testing in Fenton’s reagent. It can be clearly seen that the weight reduced with the increase of AMPS content, indicating that oxidation attacks on the membrane by radical species occurs mainly on AMPS units. Chemical stability is an essential requirement for long-term durability and maximum weight % loss in acid (AT) 3.78 and in base (BT) 2.55 % (Table 1) for polymer electrolyte. In addition, we also studied proton conductivity after the chemical stability test.

### 4.6. Proton Conductivity and Activation Energy

**Scheme 3:**
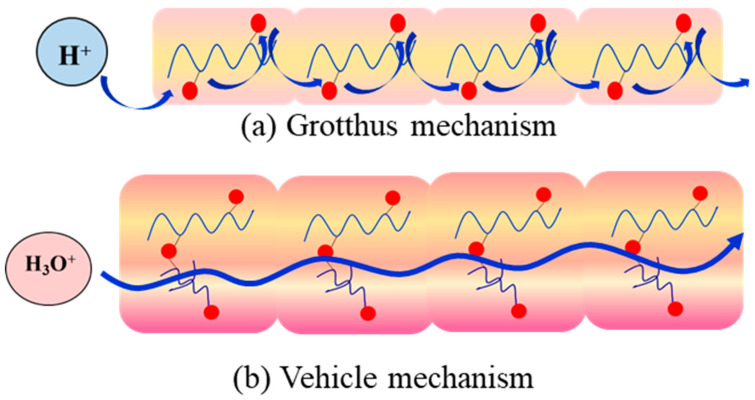
Proton transfer mechanism in SPEES and SPEES-AM membranes: (**a**) Grotthus mechanism (**b**) vehicle mechanism.

Immobile sulfonic acid groups may dissociate and the hydronium ions (e.g., H_3_O^+^, H_5_O_2_^+,^ and H_9_O_4_^+^) were formed via hydrogen bonding around the sulfonic acid groups from sulfonated polymer electrolytes in a hydrated condition [30,31]. In swollen state, free protons easily transport from sulfonated polymer electrolyte membranes along with the hydrogen-bonded ionic network so that the proton conductivity will be clearly enhanced by the water uptake in the membrane matrix. Proton conductivity is one of the most important properties for PEMFC membranes. The proton conductivity is discussed with the following mechanism [25], presented in Scheme 3a Grotthus or “jump” mechanism which can be idealized as protons being passed down the chain of water molecules and ion exchange sites; and (b) vehicle mechanism which assumes that protons combine with solvent molecules to yield complexes like H_3_O^+^ and then diffuse as a whole across the membrane. We observed that the high conductivity of SPEES-AM membranes is due to the linked ionic cluster structure as proved by SAXS and SEM analysis formed in the membrane. Ionic cluster such as SPEES-AM have not been found in the SPEES membrane to allow H^+^ to jump from one –SO_3_H group to another through the channel in the presence of water. In the SPEES-AM membranes, on the other hand, the H^+^ transfer had to be facilitated with the tertiary (3°) amine and –SO_3_H groups to form ionic bonds.

**Figure 9:**
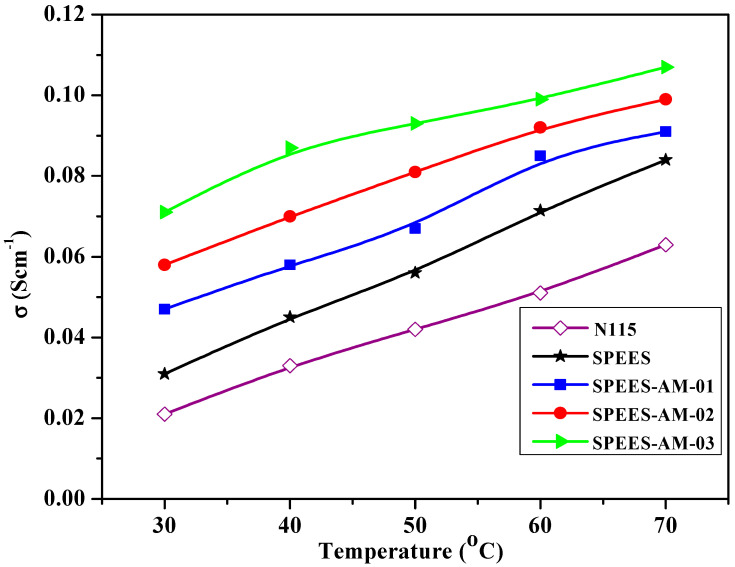
Proton conductivity of Nafion (N115), SPEES and SPEES-AM membranes.

As presented in Figure 9, the proton conductivity of SPEES-AM membranes are dependent on the water uptake and sulfonic acid group content in the membrane sites. It should be noted that the proton conductivity of SPEES-AM-01 showed 0.047 Scm^−1^ while SPEES-AM-03 showed 0.071 Scm^−1^ in acid form at room temperature and Nafion N 115 showed 0.021 Scm^−1^ under the same measurements. In this work the observable point is the increase of AMPS monomer because of the enhancement of water uptake and proton conductivity. It can be clearly seen that the conductivity SPEES-AM-01 exhibited ~ 0.09 Scm^−1^, SPEES-AM-02 ~ 0.094 Scm^−1^, and SPEES-AM-03 ~ 0.13 Scm^−1^ which are relatively comparable in proton conductivity with temperature increased at 80 °C and 90% RH. SPEES-AM-02 and SPEES-03 were not stable up to 80 °C due to high water-swelling in membrane matrix on the effect of high ionic sites (SO_3_H).

**Figure 10:**
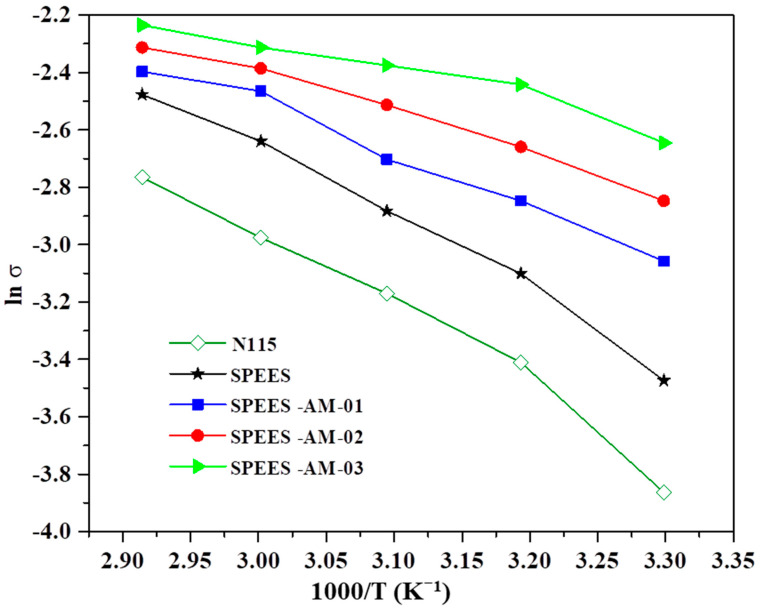
Arrhenius plot of Nafion (N115), SPEES and SPEES-AMs.

In polymer electrolyte membranes, the activation energy is an important parameter for proton conduction that takes the minimum energy part for proton transfer. The activation energy for proton conduction must be reduced which can reduce the energy loss produced that would be favorable for refining energy consumption during fuel cell operation. The activation energy is obtained based on the proton conductivity dependent on time and temperature. Protons move rapidly during high temperatures in a solids material, which follows a simple Arrhenius law and is shown in Figure 10. The activation energy (Ea) was determined from the temperature dependence of ion conductivity applying the Arrhenius Equation (5).

(5) $$\ln\;\sigma \;=\;\ln\;\sigma_{0}\;-\;\frac{E_{a}}{RT}$$

**Figure 11:**
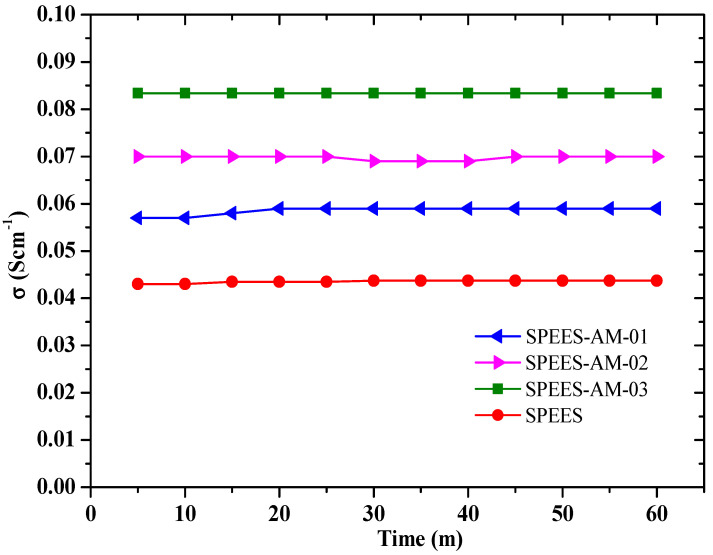
Proton conductivity of SPEES and SPEES-AM membranes at 45 °C for an hour after acid stability.

The proton conductivity is also checked at constant temperature (45 °C) after acid stability up to an hour in Figure 11 and all membranes observed that the conductivity is also constant. We have seen that all membranes perform well and there is no one membrane that loses its conductivity property. Therefore, SPEES-AMs are completely sufficient for fuel cell operations at low temperature with low RH. Continuity is not seen beyond 65 °C conductivity and has little fluctuation and also affects the backbone of the membrane. The water uptake of SPEES-AM membranes upward with the increasing AMPS content, which loosens the compact structure of membrane, and therefore some sulfonic acid groups leach into the water during the measurement. It should be mentioned that none of the membranes were broken into small pieces, and all samples remained in a good membrane form after the 120 h test period.

### 4.7. Fuel Cell Performance

**Figure 12:**
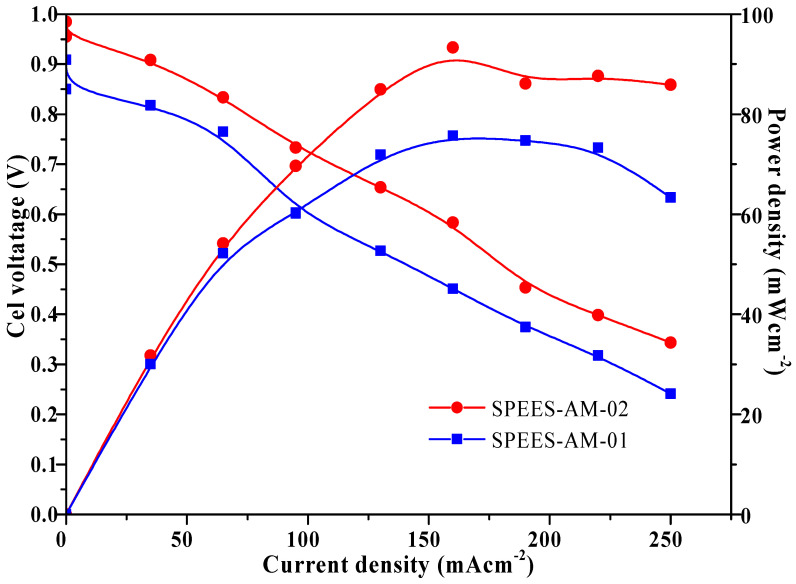
Fuel cell performance of SPEES-AM membranes in H_2_/ O_2_ single cell PEMFC test at 50 °C (under 15% RH).

The H_2_/O_2_ fuel cell performance of SPEES-AM membranes is shown in Figure 12. As shown in Figure 12 SPEES-AM-01 and SPEES-AM-02 exhibited the open-circuit voltage (OCV) of 0.90 V and 0.98 V respectively. The fuel cell performance has been tested at 50 °C under 15% RH. This research will create a new achievement fuel cell operation at low temperature with RH and utilization of water molecule generated at the cathode section. SPEES-AM-02 showed a maximum power density of 92 mWcm^−2^ at a current density of 159 mA cm^−2^, while SPEES-AM-01 showed a peak power density of 75 mWcm^−2^. Therefore, it seems that the fuel cell operation at low temperature can facilitate operation and energy in order to reduce the energy consumption and can facilitate in the field of energy.

## 5. Conclusions

A hybrid concept based on aromatic and aliphatic polymer electrolyte membranes with synthesized SPEES and electrolyte monomer AMPS exhibits elevated proton conductivity of 0.125 Scm^−1^ at 70 °C (100% RH). The presence of AMPS in the SPEES domain enables to provide high electrolyte. The water uptake and swelling behavior of the membranes are key factors that significantly affect the proton conductivity, and mechanical properties of the membranes. The AMPS-based membrane deteriorated at high temperature (above 65 °C) because of high functional charge with sulfonic, tertiary amine and ketone. All these membranes have been shown to have constant conductivity during constant temperature (45 °C) and can be of great benefit at low temperatures under low RH for fuel cell applications with cost-effectiveness.

## Figures and Tables

**Table 1:** Proton conductivity (σ), water uptake (WU), swelling ratio (SR), acid-base test (AT-BT), and IEC of SPEES and SPEES –AM Membranes at 30 °C.

Membrane	σ	WU (%)	SR (%)	AT (%)	BT (%)	IEC (meq·g^−1^)
SPEES	0.031	16	20	3.25	4.11	1.75
SPEES-AMPS-01	0.047	20	27	0.11	0.21	1.87
SPEES-AMPS-02	0.058	26	35	1.22	0.45	2.01
SPEES-AMPS-03	0.071	30	41	3.78	2.55	2.17
N115	0.021	11	13			

**Table 2:** Solubility data.

Solvent	PEES	SPEES	SPEES-AMPS
CH_3_OH	Insoluble	Insoluble	
CH_3_OCH_3_	Insoluble	Insoluble	
DMAc	Insoluble	Soluble at <40 °C	Soluble at 25 °C
DMF	Insoluble	Soluble at <40 °C	Soluble at 25 °C
THF	Insoluble	Insoluble	Soluble at 45 °C
NMP	Insoluble	Soluble at 50 °C	Soluble at 25 °C
DMSO	Insoluble	Soluble at 50 °C	Soluble at 25 °C
H_2_SO_4_	Soluble	Soluble	Soluble
